# Impact of the innate environment on maintaining memory T-cell numbers in the female genital tract: implications for mucosal vaccine efficacy?

**DOI:** 10.1186/1742-4690-9-S2-P193

**Published:** 2012-09-13

**Authors:** S Dabee, J Passmore, A Williamson, P Gumbi

**Affiliations:** 1 University of Cape Town, Cape Town, South Africa

## Background

Preventative HIV vaccines aim to elicit long-lived protective immune responses at the site of HIV transmission, capable of responding quickly to HIV challenge, but which remain stable at effector sites of the genital mucosa. The genital mucosa is, however, commonly confronted with innate immune modifiers and inflammatory agents including sexually-transmitted infections, behavioural and hygiene practices. We investigated the impact of mucosal inflammation and homeostatic cytokines on local T-cell phenotype, proliferation, exhaustion and activation.

## Methods

Levels of activation (CD38, HLA-DR), proliferation (Ki67) and senescence (CD57) were measured on T-cells isolated from cervical cytobrushes and blood from 46 HIV-negative women by flow cytometry and inflammatory cytokines and IL-7 in genital secretions were measured by ELISA.

## Results

HIV-negative women generally had higher concentrations of inflammatory (IL-1β, IL-6, IL-8) than homeostatic cytokines (IL-15, IL-7) in their genital secretions. Cervical IL-7 correlated positively with inflammatory cytokine concentrations, suggesting that inflammation and homeostatic cytokine production were linked. HIV-negative women with lower cervical CD4^+^ T-cell frequencies had the highest concentrations of genital IL-7 (p=0.028; rho=-0.23) suggesting that local IL-7 production increased in response to elevated CD4^+^ T-cells. In vitro culture of T-cells with IL-7 caused an increase in activation of both CD4^+^ (CD38 p=0.002; HLA-DR p=0.01) and CD8^+^ (CD38 p=0.01; HLA-DR p=0.006) T-cells compared to cells not stimulated with IL-7. IL-7 also caused an increased Ki67 expression by CD4^+^ T-cells (p=0.04).

## Conclusion

In conclusion, local IL-7 in the presence of genital inflammation may favour T-cell activation and turnover of vaccine-induced responses in the genital tract.

